# Post-pandemic influenza A (H1N1) 2009 virus infection in pregnant women in Ceará, Brazil

**DOI:** 10.1111/irv.12347

**Published:** 2015-10-13

**Authors:** Anne C B Perdigão, Fernanda M C Araújo, Maria E L Melo, Daniele R Q Lemos, Luciano P Cavalcanti, Izabel L C Ramalho, Lia C Araújo, Deborah M Sousa, Marilda M Siqueira, Maria I F Guedes

**Affiliations:** a Central Public Health Laboratory of CearáFortaleza, Brazil; b Northeast Biotechnology Network, State University of CearaFortaleza, Brazil; c Christus University Center, UnichristusFortaleza, Ceará, Brazil; d State Health Secretariat of CearáCeará, Brazil; e University Federal of CearaCeará, Brazil; f University of Fortaleza – UNIFORCeará, Brazil; g Oswaldo Cruz Institute/IOC/FIOCRUZRio de Janeiro, Brazil

**Keywords:** Placental transmission, post-pandemic influenza A, pregnant

## Abstract

**Objective:**

The aim of this study was to present results of the post-pandemic phase of A(H1N1)pdm09 virus infection in pregnant women in Ceará, Brazil, during the January–June 2012 influenza season.

**Results:**

One hundred and fifty-four nasopharyngeal swab samples were collected from pregnant women admitted to hospitals with suspected severe acute respiratory infection (SARI). Fifty-three (34·4%) had laboratory-confirmed A(H1N1)pdm09 virus infection with 15 (28·3%) outpatients and 38 (71·7%) hospitalized. Five (9·4%) women were in the first trimester of pregnancy, 20 (37·7%) in the second trimester of pregnancy, and 24 (45·2%) in the third trimester of pregnancy. Three had no information about the time of pregnancy. Six samples from newborns were also analyzed, of which three were nasopharyngeal swab positive for A(H1N1)pdm09. These swabs were collected immediately after birth, with the exception of one that was collected on the day after birth.

**Conclusion:**

Our findings suggest that transplacental transfer of influenza viruses could occur as a result of severe illness in pregnancy. It is therefore important to encourage women to be vaccinated against influenza in order to avoid pregnancy complications.

## Introduction

The pandemic influenza A (H1N1) 2009 [A(H1N1)pdm09] virus emerged in Mexico in April 2009.^1^ However, the first cases reported of this new influenza virus were in two different counties in California, USA, in April 2009; two children had respiratory infections.^2^ The A(H1N1)pdm09 virus has been identified as the cause of a worldwide outbreak of febrile respiratory infection and has been declared a pandemic disease by the World Health Organization (WHO) in June 2009.^1^,^3^ On July 19, 2009, the Ministry of Health of Brazil stated that the outbreak of the disease was a result of the sustained transmission of A(H1N1)pdm09.

Pregnancy in such cases was identified as a risk factor for developing severe A(H1N1)pdm09 virus infection. This increased risk is believed to be due to physiologic adaptations in the respiratory, cardiovascular, and immune systems. Infection with influenza during pregnancy also led to an increased risk of adverse outcomes such as preterm delivery.^5^–^7^

Pregnant women were at least four times more likely to be hospitalized than the general public when infected with this new influenza virus.^5^ In Brazil, cases of SARI caused by pandemic influenza virus were confirmed in pregnant women. Of these, 19·9% occurred in women of reproductive age, 24·1% of those outcomes being fatal in this group.^4^ In Ceará, a state located in northeastern Brazil, there were eight fatal outcomes due to pandemic influenza. Of these, three were pregnant women.^8^

On August 10, 2010, the WHO declared the beginning of the post-pandemic phase of A(H1N1)pdm09 virus.^9^ During the epidemiological phase, medical services continued to remain vigilant and maintain surveillance, especially for high-risk groups, such as pregnant women.

As the declaration of the sustained transmission of A(H1N1)pdm09, the report of SARI cases has been compulsory and influenza surveillance has continued to monitor respiratory disease activity. Nasopharyngeal swab samples have been collected from the reported cases and sent to a reference laboratory for analysis.

The aim of this study was to present the results of the post-pandemic phase of A(H1N1)pdm09 virus infection in pregnant women in Ceará, Brazil, during the January–June 2012 influenza season.

## Methods

During the January–June 2012 influenza season, 245 nasopharyngeal swab samples were collected from 154 pregnant women and 91 non-pregnant women of reproductive age group with suspected influenza such as ILI or SARI; all were attended to in hospitals. The swabs, together with the requisite research data form, were sent to the Central Public Health Laboratory of Ceará (CPHL-CE) for analysis. The CPHL-CE is the state reference laboratory for influenza diagnosis.

The pregnant women were attended to in the emergency rooms of several hospitals from 11 counties in the state: Aracati, Beberibe, Cascavel, Caucaia, Fortaleza, Horizonte, Itaitinga, Maracanau, Pacajus Paraipaba, and São Luis do Curu.

The cases were women who were pregnant at the time of the onset of symptoms of ILI or SARI. The surveillance case definitions for ILI and SARI followed the WHO recommendation: ILI case definition was an acute respiratory infection with measured fever of ≥38°C and cough with onset within the last 10 days; SARI case definition was an acute respiratory infection with the same characteristics similar to ILI, but with a need of hospitalization.^10^

Samples from six newborns were collected and analyzed to investigate the possible transplacental transmission of the influenza virus. Five babies were delivered by Cesarean section and one was a vaginal delivery. The deliveries were performed in isolated operation rooms. The infants were transferred to isolated sterile rooms in the neonatal medium care unit (NMCU).

A nasopharyngeal swab sample was collected from each infant immediately after birth, with the exception of one that was collected on the day after birth. The swabs were then sent for analysis to CPHL-CE in order to screen for A(H1N1)pdm09 virus infection. The mothers and staff with respiratory symptoms were not allowed to have any contact with the babies until the nasopharyngeal swab samples had been collected.

After collection, the samples were sent to CPHL-CE and maintained at a temperature of 2–8°C, together with the influenza research records. Upon arriving at the laboratory, the samples were processed and tested with a real-time reverse transcriptase PCR (qRT-PCR) assay that was developed at CDC.^11^,^12^ For nucleic acid extraction, the QIAamp Viral Mini Kit (Qiagen) was used for sample extraction following the manufacturer's recommended procedures.

The CPHL-CE protocol to investigate a suspected A(H1N1)pdm09 case was employed to process all the samples by qRT-PCR to detect the A(H1N1)pdm09 virus, and the negative ones were detected by an immunofluorescence indirect assay. The Respiratory Virus Panel IFA kit (Biotrin International Ltd., Dublin, Ireland) was used for making a differential diagnosis of other respiratory viruses. This test serves as an assay for screening and identifying adenovirus, influenza A and B, parainfluenza type 1, 2, and 3, and respiratory syncytial virus (RSV). The manufacturer's recommended procedures were followed.

A confirmed case was defined as an acute respiratory illness with laboratory-confirmed A(H1N1)pdm09 virus infection by qRT-PCR (including pre-admission, in-hospital, or postmortem sample).

The proportion of cases with specific manifestations, age, vaccination status, comorbidities, and clinical course was compared with the number of pregnant and non-pregnant women of reproductive age (14–49 years old). Risk ratios (RR) were calculated using standard methods (95% CI was calculated using the Taylor series method). The proportion of differential diagnosis for other respiratory viruses was calculated for pregnant women and non-pregnant women of reproductive age.

The study was approved by the Ethics Committee of the Hospital São José de Doenças Infecciosas.

## Results

From January to June 2012, CLPH-CE received nasopharyngeal swabs collected from 154 pregnant women and 91 non-pregnant women of reproductive age (14–49 years) with ILI or SARI, to perform a laboratory investigation for A(H1N1)pdm09 virus. The influenza virus was found in 35% (86/245) of the samples. A total of 53 (34·4%) pregnant women and 33 (36·3%) non-pregnant women of reproductive age had laboratory-confirmed A(H1N1)pdm09 virus infection.

Of the cases in pregnant women, 36 (67·9%) were reported by hospitals in Fortaleza, the state capital, and 17 (32·1%) by hospitals in other counties.

The ages of the pregnant women ranged between 14 and 37, the average being 24·8. Only 12 (22·6%) had previously been vaccinated against influenza. Of the 53 confirmed pregnant cases, 15 (28·3%) were outpatients and 38 (71·7%) were hospitalized. Five (9·4%) women were in the first trimester of pregnancy, 20 (37·7%) in the second trimester of pregnancy, and 25 (47·2%) in the third trimester of pregnancy. Three (5·3%) had no information about the time of pregnancy (Table1).

**Table 1:** Characteristics of women with A(H1N1)pdm09 infection, according to pregnancy status, from January to June 2012

Characteristics	Pregnant *N* (%)	Non-pregnant *N* (%)	RR	CI	*P*-value
Age (year)					
≤24	28 (52·8)	16 (36·4)	1·09	0·77–1·53	0·398
>24	25 (47·1)	17 (41·5)			
Gestational age					
1° Trimester	5 (9·4)	NA			
2° Trimester	20 (37·7)	NA			
3° Trimester	25 (47·2)	NA			
Unknown	3 (5·7)				
Vaccination					
Yes	12 (22·6)	09 (27·3)	1·20	0·79–1·84	0·255
No	29 (54·7)	13 (39·4)			
Unknown	12 (22·6)	11(33·3)			
Symptoms					
Fever	41 (65·1)	22 (34·9)	1·26	0·90–1·75	0·233
Cough	41 (66·1)	21 (33·9)	1·13	0·78–1·64	0·407
Dyspnea	38 (73·1)	14 (26·9)	1·34	0·88–2·03	0·100
Coryza	34 (66·7)	17 (33·3)	1·04	0·75–1·46	0·513
Myalgia	25 (59·5)	8 (24·2)	1·27	0·93–1·74	0·108
Sore throat	23 (62·2)	14 (38·7)	1·17	0·85–1·61	0·228
Chills	20 (60·6)	13 (39·4)	1·18	0·84–165	0·229
Diarrhea	09 (63·6)	04 (36·4)	1·07	0·66–1·73	0·507
Hospitalization					
Yes	38 (86·4)	06 (13·6)	2·39	1·52–3·75	0·000
No	15 (36·1)	27 (63·7)			
Death	01	04			

NA, not applicable; RR, relative risk; CI, confidence interval.

The most prevalent symptoms among the pregnant women with influenza virus infection were fever and cough in 41 (77·3%) cases, followed by dyspnea in 38 cases (71·7%) and rhinorrhea in 34 (64·2%) cases (Table1). Seven pregnant women had some associated comorbidities such as asthma (three), smoking (one), lung disease (two), hypertension (one), and diabetes (one). Of them, four were admitted to the intensive care unit (ICU), two presented with asthma, one presented with diabetes, and the one that smoked died. The pregnant woman with a fatal outcome was in the second trimester of pregnancy and was admitted to the hospital presenting fever, cough, and breathlessness, which after 2 days evolved into acute respiratory distress syndrome and respiratory arrest. She was revived and transferred to the ICU where she presented kidney and liver failure and died after 1 day. Fetal death occurred at the same time as maternal death. All patients were treated with oseltamivir, after the samples had been collected.

The differential diagnosis with other respiratory viruses was performed in 101 negative samples of A(H1N1)pdm09 virus from pregnant women and 58 non-pregnant women of reproductive age. It was found that 25% of the pregnant women were positive for other respiratory viruses, 16% for adenoviruses, 4% for RSV, 3% for influenza B, and 1% for parainfluenza 1 and 3. In the group of non-pregnant women, 19% were positive for other respiratory viruses, 13% for adenoviruses, and 3% for influenza B and parainfluenza 2.

Six samples from newborns were also analyzed, of which five were preterm Cesarean deliveries and one was a vaginal delivery. The samples were collected immediately after birth, before any contact with the mothers. The one that was born during the night had a swab taken the following day, again before any contact with the mother. Of these, three newborns were nasopharyngeal swab positive for A(H1N1)pdm09, two of them had mild dyspnea, and one did not show any symptoms. All the newborns were transferred to an isolated sterile room; however, none needed admission to the ICU, and they were kept in incubators in the NMCU (Table2). The three infants with influenza infection were treated with oseltamivir.

**Table 2:** Characteristics of pregnant women with A(H1N1)pdm09 who were hospitalized and delivered during infection period

Patients	Age (years)	Gestational age at admission	Clinical and delivery-related characteristics	Treatment with oseltamivir	Pregnancy outcome	Neonatal influenza infection
01	23	3° trimester	Fever, cough, dyspnea, labor induced because of preeclampsia and Cesarean delivery due to acute respiratory syndrome	Yes	Live birth	No
02	17	3° trimester	Cough, sore throat, and runny nose 3 days; vaginal delivery	Yes	Live birth	No
03	14	3° trimester	Fever, cough, chills, dyspnea, sore throat, arthralgia, myalgia, runny nose, and diarrhea 5 days before preterm labor and Cesarean delivery	Yes	Live birth	Yes
04	19	3° trimester	Fever, cough, dyspnea; Cesarean delivery	Yes	Live birth	No
05	37	3° trimester	Fever, cough, runny nose; Cesarean delivery with 2 days of onset of symptoms; smoking patient	Yes	Live birth	Yes
06	33	3° trimester	Fever, cough, dyspnea, sore throat, myalgia, runny nose, and diarrhea; preterm Cesarean delivery with 4 days of onset of symptoms	Yes	Live birth	Yes

## Discussion

During influenza epidemics, pregnant women constitute a high-risk group related to morbidity. There is also an increased risk of adverse pregnancy outcomes, as seen during the pandemic H1N1 influenza virus, in 2009–2010.^5^,^6^,^13^–^15^ In Ceará, during the 2009–2010 influenza pandemic phase, pregnant women accounted for about 37·5% of the deaths due to confirmed A(H1N1)pdm09 virus infection.^8^ This might explain why there were a greater number of reported cases among pregnant women than among non-pregnant women of reproductive age, as there was no significant difference among the positivity of the two groups.

The risk of receiving a clinical diagnosis of influenza is reduced when women are vaccinated during pregnancy.^16^ Pregnant women should be given the influenza-inactivated vaccine due to the increased risk of illness and death during pandemic influenza.^5^,^6^,^16^,^18^ Although influenza vaccination is safe, only 22% of pregnant women were vaccinated. Similar low numbers have been reported in other studies.^5^,^17^,^19^

This could be explained by the fact that vaccination during pregnancy had not been recommended by the physician, due to both the doctor and the pregnant woman's fears over the safety of the vaccine.^17^

The risk of severe illness among pregnant women is significantly higher for those in the second and third trimesters of pregnancy.^20^ Regardless of their gestational age, most of the women were in the second (37·7%) and third (47·2%) trimesters; only 9·4% were in the first trimester of pregnancy. This seems to be a common finding worldwide.^5^,^6^,^20^–^23^

The immunologic changes in pregnancy might have induced a state of increased susceptibility to some intracellular pathogens such as viruses and intracellular bacteria and parasites.^24^ The results of a study conducted in New York in 2010 showed that pregnant women were 7·2 times more likely to be hospitalized with pandemic influenza than non-pregnant of reproductive age, the hospitalization rate being 4·3 times higher for severe cases.^13^ We found that pregnant women were 2·39 times more likely to be hospitalized than non-pregnant women of reproductive age.

The increased hospitalization of pregnant women was possibly due to the provision of greater care in view of the risk of severe infection with A(H1N1)pdm09 virus during the pandemic period (from April 19, 2009, to November 18, 2010, in our state).^8^

We found eight comorbidities in seven (13%) pregnant women with SARI. These findings suggest that the pregnancy itself, rather than the comorbidities, was responsible for the clinical course of the influenza.

The effects of seasonal and pandemic influenza on the fetus are not well understood. In this study, three infections were reported in infants born to women with A(H1N1)pdm09 virus infection; two of them were suggestive of transplacental infection because the swabs were taken immediately after Cesarean section and before contact with the mothers. One newborn had the swab collected on the day after birth, so we cannot ensure whether he was infected before his birth.

A case–control cohort study identified influenza virus infection in 182 of 1659 (11%) pregnancies; however, there was no evidence of transplacental transmission of the influenza virus.^22^ A study of placental tissues carried out in seven women with confirmed H1N1 infections did not show any evidence of placental transmission.^25^

Although placental transmission seems to be rare, it has been observed in pregnant women infected with H5N1 and in a fatal case of influenza during the third trimester of pregnancy.^22^,^26^ There are some case reports on suspected transplacental transmission of A(H1N1)pdm09 virus from the mother to the amniotic fluid and fetal heart.^27^

A retrospective investigation of the patient's products of conception identified subtype H1N1, resulting in a second trimester loss.^15^ In Thailand, a newborn girl, whose mother became infected with the A(H1N1)pdm09 virus, was confirmed with influenza.^28^ In Peru, an indigenous female neonate that had a throat swab collected within 5 hours of life was confirmed positive for the A(H1N1)pdm09 virus by RT-PCR.^29^

A newborn male was diagnosed with congenital pneumonia and then confirmed positive for the A(H1N1)pdm09 virus, although there was no known contact with an infected person.^30^ In addition, a female premature infant, whose mother had been confirmed for A(H1N1)pdm09 virus infection, was born at 32 weeks of gestation and then confirmed positive for the A(H1N1)pdm09 virus.^31^

## Conclusion

Our findings suggest that transplacental transfer of influenza viruses is possible. Severe illness occurred in pregnancy, especially in the second and third trimesters. Therefore, it is important to encourage pregnant women to be vaccinated against influenza in order to avoid pregnancy complications.
